# Amy Blevins, Medical Library Association President, 2023–2024

**DOI:** 10.5195/jmla.2025.2089

**Published:** 2025-01-14

**Authors:** Elizabeth Kiscaden, Hannah J. Craven, Gabriel R. Rios, Ryan Harris, Joey Nicholson

**Affiliations:** 1 elizabeth.kiscaden@uc.edu, Dean and University Librarian, University of Cincinnati, Cincinnati, OH; 2 hancrave@iu.edu, Associate Librarian, Ruth Lilly Medical Library, Indiana University School of Medicine, Indianapolis, IN; 3 grrios@iu.edu, Library Director, Ruth Lilly Medical Library, Indiana University School of Medicine, Indianapolis, IN; 4 rharr103@charlotte.edu, Associate Dean for Public Services, UNC Charlotte J Murrey Atkins Library, University of North Carolina, Charlotte, NC; 5 joey.nicholson@nyulangone.org, Associate Curator, NYU Health Sciences Library, NYU Langone Health, New York, NY

## Abstract

Amy Blevins served as the Medical Library Association president from 2023–2024. In this presidential biography, the authors outline a history of Blevins' recruitment to the career, career development, and impact on the association and the profession.

**Figure:**
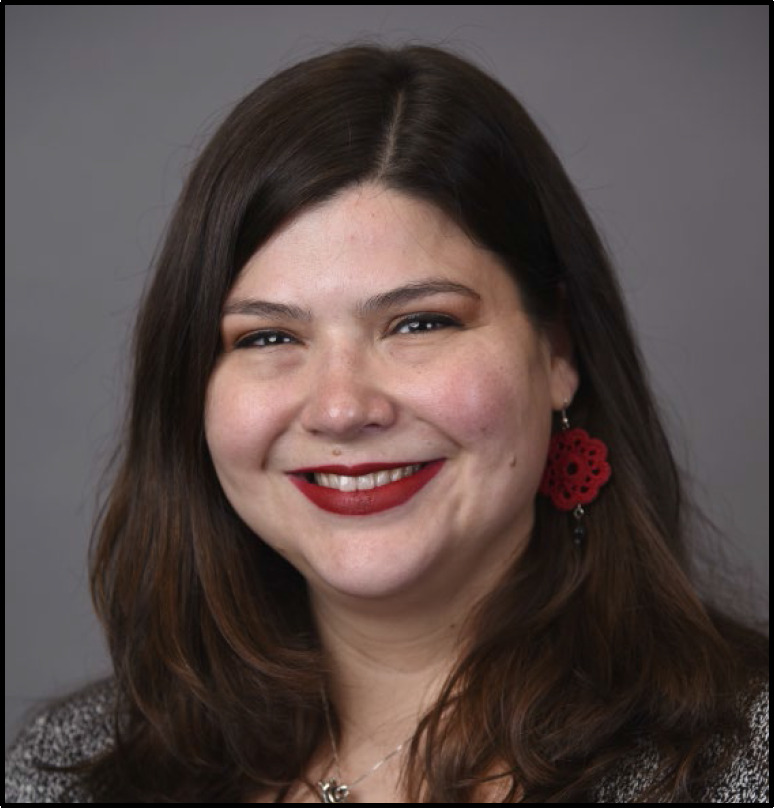


Amy Blevins' colleagues, mentors, and friends always knew it was not a matter of if, but rather when she would serve as the president of the Medical Library Association. Enthusiastic, driven, highly intelligent, and deeply engaged in the profession, Blevins stands apart for her passion for health sciences librarianship and commitment to advancing the profession. What is more, throughout the highs and lows of building our association or progressing in her own career, Blevins makes it fun. Have you ever served on a committee with Blevins? If you did, you would remember it, as she likely had you laughing!

Don't let Blevins' animated nature or unconventional interests lead you to underestimate her professional acumen. In your acquaintance with Blevins, she may have educated you about the harmless, yet slimy, hellbender salamander, which is a threatened aquatic species. Or, given her love for horror movies, she may have shared the knowledge that the movie *Critters* was based on a nightmare the producer had when he was a child. While discussing the cryptids of North America or admiring her signature goth earrings, do not forget that Blevins is a research powerhouse and one of the most skilled instructors of evidence-based practice in our field. Blevins has a commitment to staying true to who she is and demonstrates how to be a professional at the top of the field on her own terms.

## RECRUITMENT TO THE PROFESSION

Blevins began her undergraduate journey at Saint Louis University where she pursued a Bachelor of Arts in Biology. She was already familiar with the university; her mother had gone back to school when Blevins was a teenager. Given that she was a single mother, she brought Blevins along to study in the library. As an incoming college student, Blevins applied for a job in the library, but was soon overachieving - early evidence of the internal drive that continues to motivate her throughout her career. It wasn't long before Blevins was promoted to an assistant role, supporting the reference services staff in the library.

Blevins' career plan was to continue on to graduate school to earn an MD/PhD in order to work as a genetic researcher, an idea inspired by a middle school program designed for academically talented students. It was thanks to the influence of many outstanding librarians she crossed paths with at the library that she changed course. She had many great supervisors and mentors at the Pius XII Memorial Library including (but not limited to): Jeannette Pierce, Martha Allen, Jamie Emery, Jonathan Harms, Phill Barron, and Claudia DuVall. Blevins came to find the academic library a second home and found a strong sense of belonging among the academic librarians who worked there-she had found her people.

Blevins went on to complete her Master of Arts in Library Science at the University of Missouri - Columbia, where she continued to work in the academic library. In fact, this is where she first gained experience working in a health sciences library, where she completed a stint as a graduate library assistant at the J. Otto Lottes Health Sciences Library. Although she originally intended to become a science librarian, her experience as a graduate library assistant solidified her intent to specialize in health sciences librarianship. It was at J. Otto Lottes that Blevins found her calling, and thanks to supportive mentors, such as Rebecca Graves, Amanda McConnell, and Diane Johnson, that she found a passion for Medical Subject Headings (MeSH) and advanced search strategies in biomedical databases.

## EAST CAROLINA UNIVERSITY

As a newly minted graduate, Blevins accepted a position as a librarian liaison to the College of Allied Health Sciences and College of Health and Human Performance at East Carolina University (ECU) in 2006. The role matched her interest in instruction (inspired by Rebecca Graves) and she set her sights on developing online instructional materials at an institution that prided itself on being the largest provider of distance education in the state at that time. During her tenure at ECU, Blevins partnered on the development of a new web page to display electronic resources, led an evaluation of online course content, and conducted an evaluation of software tools used for video tutorials.

**Figure 1:**
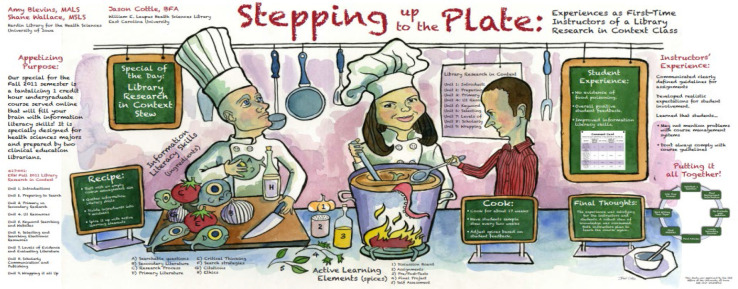
Amy Blevins, S. Wallace, & J. Cottle. “Stepping Up to the Plate: Experiences as First-Time Instructors of a Library Research in Context Class.” Medical Library Association Annual Conference, 21 May 2012.

It was through Blevins' work designing and delivering online instruction and creating tutorials that she moved into the newly created position of Education and Instructional Technology Librarian at ECU. Developing herself further in this role, Blevins designed longitudinal curriculum-based instruction for an occupational therapy program, which had an evidence-based practice component. She went on to lead the Tea Time Training program for training staff internally and to host a podcast with the main academic library titled, *Research First Aid*. It is worth noting that Blevins' research posters from this chapter of her life feature outstanding original art. AtECU, Blevins partnered with graphic designer, Jason Cottle, who went on to become a lifelong personal friend. Due to this connection, research posters from this time period feature animated scenes, such as a kitchen in which the copresenters are featured chopping up the “ingredients” for information literacy skills (see Figure 1). The “active learning elements” are ready to pitch into the cooking pot, and from within that pot, a long, pink squid tentacle can be seen, draping over the edge [1].

Blevins often acknowledges the William E. Laupus Library at ECU for supporting her early engagement in the profession regionally and nationally. The library provided professional development funds which allowed Blevins to attend the Medical Library Association's Mid-Atlantic Chapter (MAC) meetings, where she quickly got involved in the organization and made lifelong connections. It was her engagement in MAC that connected her with Shannon Jones, another former MLA president and a legend within the profession of health sciences librarianship. Jones was Blevins' conference mentor at her first health sciences librarian conference and has mentored her throughout her career.

## UNIVERSITY OF IOWA

Seeking to further develop herself, Amy Blevins accepted a Clinical Education Librarian position at Hardin Library for the Health Sciences at the University of Iowa. Within this role, she served as the liaison to the School of Medicine and received a faculty appointment within the Carver College of Medicine. Blevins wasted no time in establishing herself in the new role, catalyzing her colleagues and expanding her professional network throughout the upper Midwest.

As a part of her role, Blevins was responsible for managing the Simulation Center, which was housed within and managed by the health sciences library. Under her leadership, Blevins collaborated with colleagues to expand a program which used simulation equipment as a tool for outreach to area school-aged children. Children from grades 2 to 12 would visit the Simulation Center to try tying surgical knots, use the eye simulators, use three-dimensional anatomy tools, and try out “Harvey,” the cardiac rhythm simulator located within the Center.

Another job responsibility assigned to Blevins was utilizing technology to improve library services. These were relatively early days for online library instruction; Blevins built on her prior experience to collaborate with colleagues on the development of best practices, policies, and procedures for the creation of online instruction. With a multi-library team, Blevins evaluated software tools to use, developed standard opening and closing screens for tutorials, and presented training sessions to library staff interested in creating their own content.

In 2011, Hardin Library's director, Linda Walton, talked about a new potential service for supporting systematic reviews. After attending the University of Pittsburgh workshop, Blevins started supporting researchers and residents. As demand for the service grew, Blevins worked with her colleagues to launch a formal systematic review service at Hardin Library. After seeking solutions from colleagues on Medlib-L (the Medical Library Association's listserv), she developed a Memorandum of Understanding (MOU), at a time when there were few examples available. She shared her library's work with others and MOUs continue to be used to ensure librarians' contributions are recognized through authorship.

**Figure 2:**
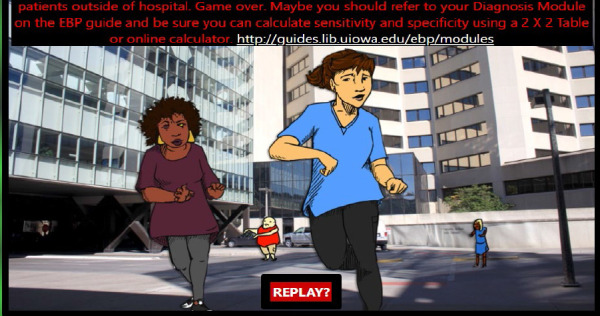
Zombies Ate My Evidence. 2017.

Back when the zombie television show, *Walking Dead*, was popular, Blevins partnered with her colleague (and coauthor of this manuscript), Elizabeth Kiscaden to develop and pilot a computer game designed to teach medical students critical appraisal skills. Combining Blevins' love of zombies with Kiscaden's love of nineties-era adventure computer games, the team used an internal grant to launch a story-based apocalypse game [2]. The game lives on (https://www.lib.uiowa.edu/hardin/zombies-ate-my-evidence/) with timeless art featuring zombies in multiple locations across the University of Iowa Health Center (see Figure 2).

## INDIANA UNIVERSITY SCHOOL OF MEDICINE

The next step in Blevins' career led her to her current role as the first Associate Director for Public Services at the Ruth Lilly Medical Library (RLML) at the Indiana University School of Medicine (IUSM). Since 2015, Blevins has made significant strides in aligning the library's public services, education, and outreach programs with the school's mission, including leading an evidence-based medicine (EBM) thread within the School of Medicine and developing a multi-tiered systematic review service.

One of Blevins' most significant contributions in this area is her work designing and implementing EBM instruction for medical students. Recognizing the importance of integrating EBM skills throughout the medical curriculum, Blevins advocated for and created a scaffolded approach to teaching these skills. This method, inspired by constructivist learning theory, ensures that students build upon their knowledge and abilities as they progress through their medical education, preparing them to be effective, evidence-based practitioners.

Typically, the leadership of major curricular components in medical schools, such as EBM education, is entrusted to clinicians who have hands-on experience in applying these principles in patient care. However, Blevins was appointed as the leader of the EBM thread at IUSM. This appointment represents a significant departure from the norm and this exceptional recognition of Blevins' expertise in teaching EBM highlights the unique and valuable contributions that librarians can make when integrated into medical education courses.

As the EBM thread leader, Blevins took on the crucial responsibility of guiding an interdisciplinary team of clinicians, biostatisticians, and other healthcare professionals, each bringing their unique perspectives and expertise to the table. Under her leadership, they developed a comprehensive scaffolded EBM curriculum that was linked back to the institutional learning objectives for the medical school. This curriculum is designed to ensure that students acquire the necessary skills to practice evidence-based medicine effectively and efficiently

Another significant contribution to the Ruth Lilly Medical Library (RLML) and the broader research community at Indiana University School of Medicine has been her creation and leadership of a formalized Systematic Review service. Recognizing the growing importance of systematic reviews in evidence-based healthcare and building on her experiences and Iowa's Hardin Library, Blevins took the initiative to create and lead a taskforce dedicated to formalizing the library's systematic review services. This move was particularly crucial given the time-intensive nature of systematic reviews, which typically take 12–18 months to produce, and their status as the highest level of research evidence when conducted properly.

Blevins' approach to the systematic review service emphasized the valuable role that librarians play in these high-impact publications. She ensured that librarians were positioned as integral team members, responsible for designing and executing comprehensive literature searches, writing the search methods sections of final publications, and advising research teams on the standards for conducting and reporting systematic reviews. The impact of this initiative has been significant as this marked a notable shift from previous practices, where librarians were more likely to be mentioned in the acknowledgments section rather than recognized as full collaborators.

The importance of this work extends beyond the library and even the university. By ensuring that RLML librarians are providing information to research teams about best practices for systematic reviews, Blevins is contributing to the publication of high-quality research that directly supports patient care. This aligns perfectly with the broader mission of evidence-based healthcare and underscores the critical role that skilled librarians play in the research ecosystem.

## NATIONAL LEADER IN EVIDENCE-BASED PRACTICE

Amy Blevins' evolution into a national leader in Evidence-Based Practice (EBP) is a testament to her dedication to advancing the field of health sciences librarianship. Her involvement in key initiatives and workshops has solidified her reputation as an expert in this crucial area.

Blevins' local work at RLML set the foundation for her involvement and leadership with the Critical Appraisal Institute for Librarians (CAIFL). Blevins was recruited by Marie Ascher in 2018 to serve on a steering committee to develop CAIFL, and the first cohort completed the program in the spring of 2019. Blevins serves as a small group facilitator for CAIFL, guiding participants through the intricacies of critical appraisal techniques. This role allows her to share her expertise while also learning from the diverse experiences of participants from across the country. The program has been a success in developing the next generation of EBP leaders in health sciences librarianship and plans are underway to offer the institute again in 2025.

Another significant milestone in Blevins' development as a national EBP leader was her involvement in the Evidence-Based Practice for Health Sciences Librarians Workshop (EBP for HSLs). Blevins' involvement began with her being invited to serve as a teaching fellow in 2018 and has transitioned into a leadership role for the national workshop. For the reimagined 2023 workshop, Blevins served on the steering committee and collaborated with a co-facilitator and teaching fellow to create materials for both large and small group sessions. Blevins independently created a new introduction to critical appraisal, including an innovative video component accompanied by a quiz for pre-work.

Blevins' work in these national initiatives extends beyond the workshops themselves, the content developed influences local practices at institutions across the country. Through these foundational workshops, Blevins has played a pivotal role in developing the next generation of medical librarians, equipping annual cohorts with essential EBM skills that they can carry forward into the profession. Blevins' work not only advances the field but also sets a standard for how EBP can be taught and implemented in library and medical education settings. As she continues to contribute to these national initiatives, Blevins is shaping the future of EBP in health sciences librarianship, ensuring that librarians remain at the forefront of evidence-based healthcare education and practice.

## LEADERSHIP IN PROFESSIONAL ASSOCIATIONS

Blevins' career is marked by a strong commitment to professional service and leadership. Her dedication to service started early on in her career when she served as chair of the MAC's Membership and Recruitment Committee. This was just the beginning of her varied and wide-ranging service to medical librarianship. Her dedication to the Medical Library Association (MLA) stands out as particularly impactful. Blevins' most recent service for the Medical Library Association (MLA) was as MLA President from 2022–2025. Before this, Blevins served as a member of the executive board and treasurer from 2016 to 2019, playing a crucial role during a period of significant change for the organization. Her leadership in financial matters, including the creation of a new Finance Committee as the MLA Treasurer, helped steer the MLA through this transitional period.

Blevins' leadership extends to various sections within the MLA, including her work with the Educational Media and Technologies Section (EMTS), for which she served in multiple leadership roles, including as chair. Under her guidance, EMTS developed free continuing education opportunities for members, an initiative that was recognized with the Section Project of the Year award. This project exemplifies Blevins' commitment to making professional development accessible to all librarians in her field.

Blevins' work for the profession has been recognized in several different ways, including being awarded the Lucretia W. McClure Award for Teaching Excellence and, with several colleagues, the Ida and George Eliot Prize [3]. While Blevins has served in many capacities for MLA, she always takes the time to foster the growth of new health sciences librarians. Blevins consistently serves as a mentor for first time conference attendees through MLA's Colleague Connection program.

Blevins' leadership abilities have been recognized beyond her immediate professional circles. Her selection for the NLM/AAHSL Leadership Fellows Program, a highly competitive program designed to prepare librarians for director positions, speaks to her potential for top-level leadership in academic health sciences libraries. At the program's capstone, Blevins was selected to give the keynote talk on behalf of the fellows. This experience has undoubtedly shaped her approach to management and strategic planning in her current role.

## LOOKING BACK, LOOKING FORWARD

Blevins' impact on the profession includes over 45 presentations, 30 journal articles, and four books or book chapters - evidence of a highly productive professional career [4]. Beyond these scholarly contributions, Blevins has developed and presented more than 25 courses on topics ranging from evidence-based practice, literature searching for systematic reviews, online instruction, assessing information needs, and critical appraisal. Beyond being active in professional associations, as noted previously, Blevins has served on countless committees within her institution.

Something particularly notable about Blevins' scholarly productivity is her practice of continuously inviting colleagues to partner with her on projects. These invitations have provided early career librarians she mentors with an opportunity to build their CVs and brought some of her former mentors back to collaborate on scholarship. Those who have worked alongside Blevins continue to be invited back for partnership on new scholarly endeavors.

Considering that Blevins is still mid-career, one wonders what future contributions she will make to the health sciences profession. While colleagues have tried to attract her to roles in the larger sphere of academic librarianship, she remains persistently committed to the discipline of health sciences. It was in this discipline that Blevins found belonging and has been only too willing to give back to the profession.

Wherever Blevins invests the second half of her career, one can count on her contributing bold ideas, putting in the work to realize change, collaborating with colleagues, and making the entire exercise fun. In the process of interviewing Blevins' former mentors for this article, authors asked them to share any final memories. In several cases, interviewees stated the same thing, “I can think of several funny stories… but they are not appropriate for the article!” But all also shared a sentiment similar to this comment from Lisa Traditi, “Amy lightens the room when she's in it, being around Amy makes you feel like everything is going to be okay even when it's hard.” Given the challenges within academic librarianship and higher education, couldn't we all use more leaders like that?
